# Supplementary material for: GSR Deficiency Exacerbates Oxidative Stress and Promotes Pulmonary Fibrosis
Source: Biomolecules. 2025 Jul 20;15(7):1050. doi: 10.3390/biom15071050 (PMC12293596; doi:10.3390/biom15071050)
Supplement: Supplementary file 1 [file biomolecules-15-01050-s001.zip › biomolecules-3719180-supplementary.pdf]

Supplementary figures

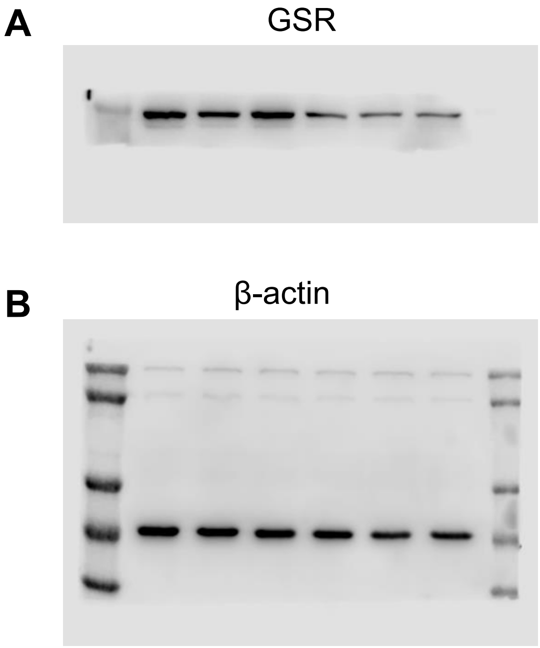

Figure S1. The original WB images of Figure 1C.

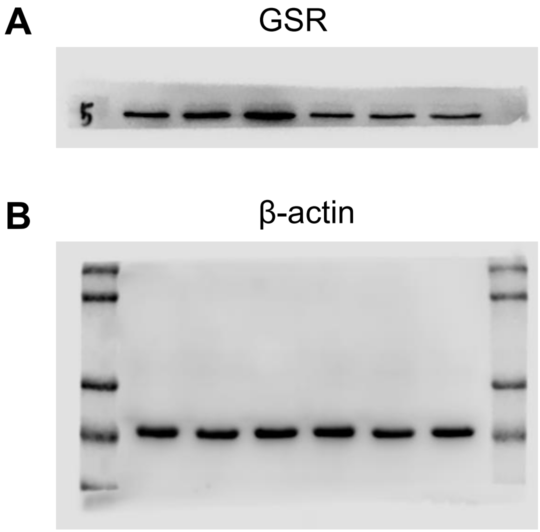

Figure S2. The original WB images of Figure 1E.

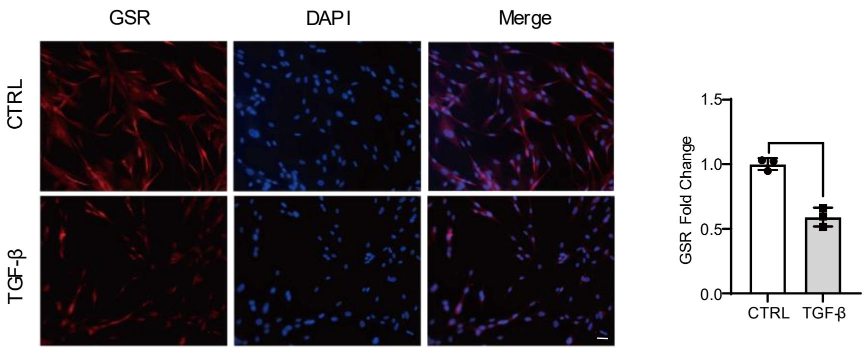

Figure S3. MRC5 cells were treated with TGF-β for 48 hours. Detecting the changes of GSR expression by immunofluorescence. The right is its quantitative statistics. n=3. Bar = 20μM. **p<0.01.

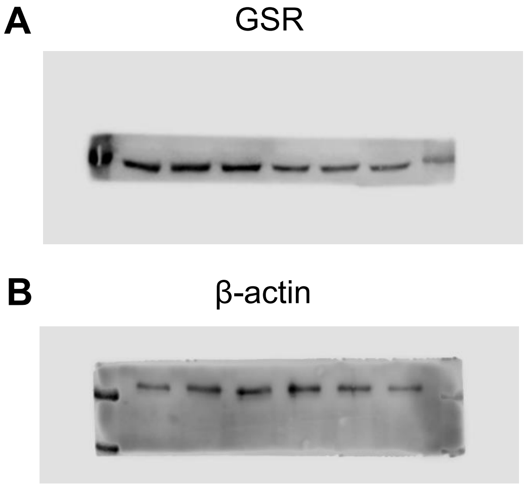

Figure S4. The original WB images of Figure 1I.

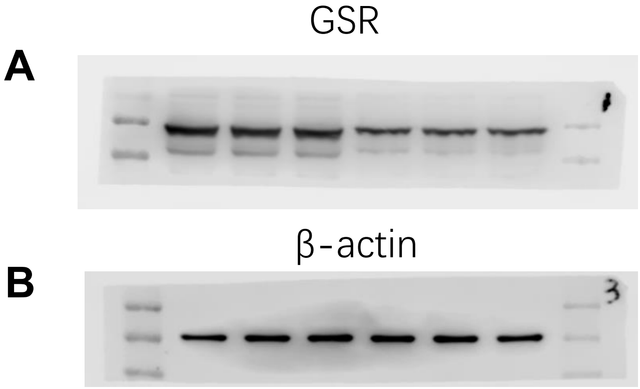

Figure S5. The original WB images of Figure 1K.

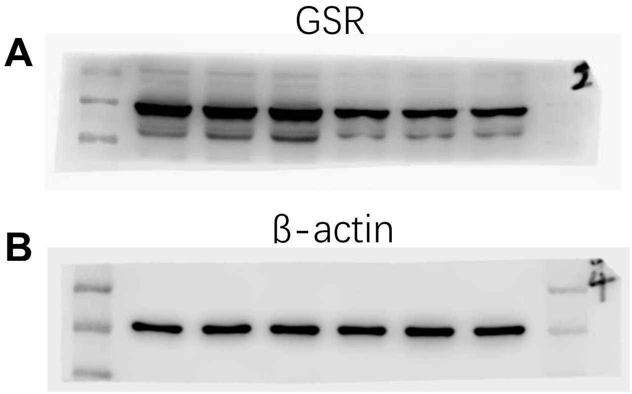

Figure S6. The original WB images of Figure 1M.

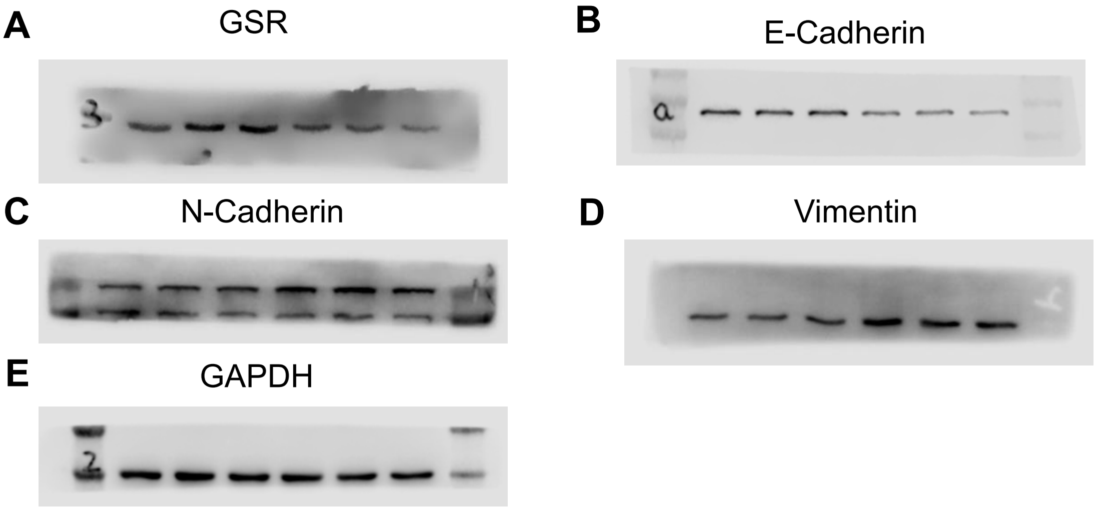

Figure S7. The original WB images of Figure 2E.

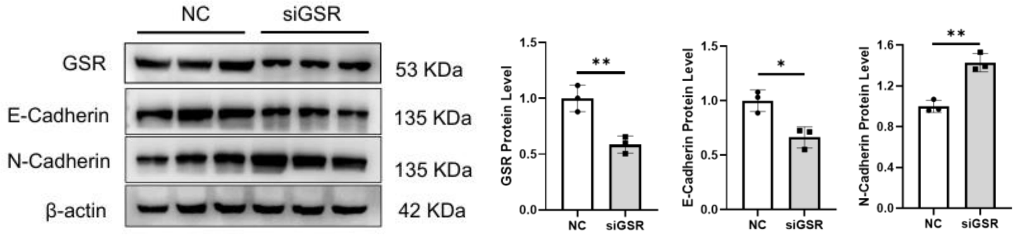

Figure S8. The expression of GSR, E-Cadherin, N-Cadherin, and β-actin in MLE-12 cells were detected by western blot. The right is its quantitative statistics. n=3. *p<0.05， **p<0.01.

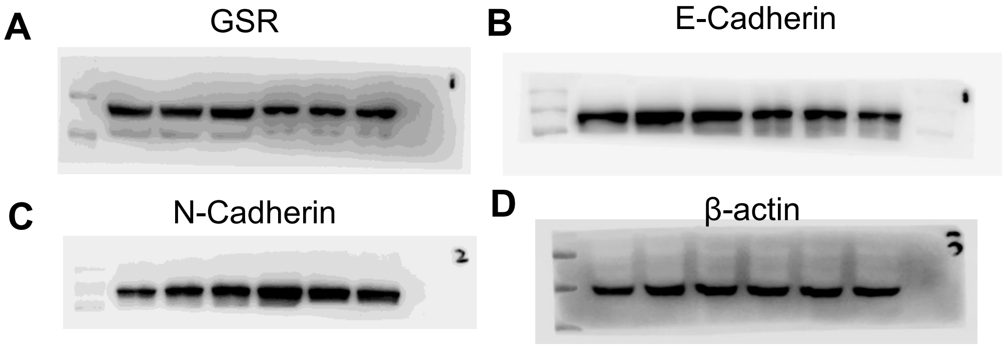

Figure S9. The original WB images of Figure S8**.**

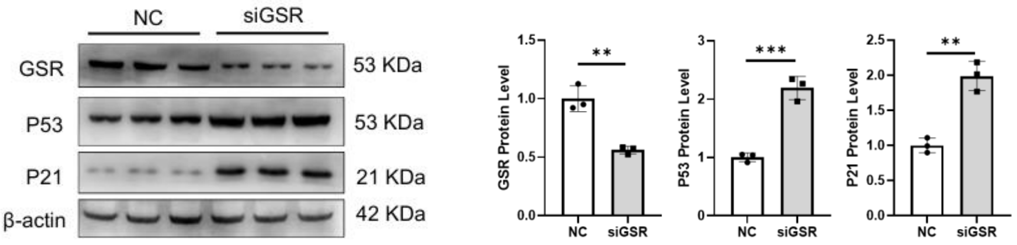

Figure S10. The expression of GSR, P53, P21, and β-actin in MLE-12 cells was detected by western blot. The right is its quantitative statistics. n=3. **p<0.01, ***p<0.001.

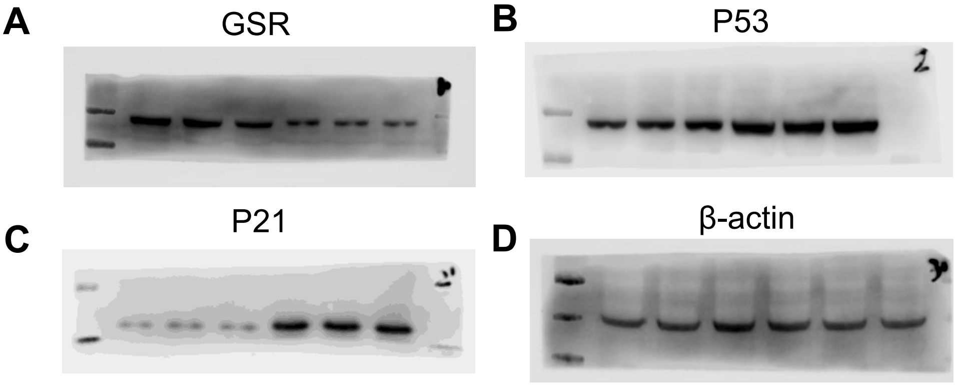

Figure S11. The original WB images of Figure S10**.**

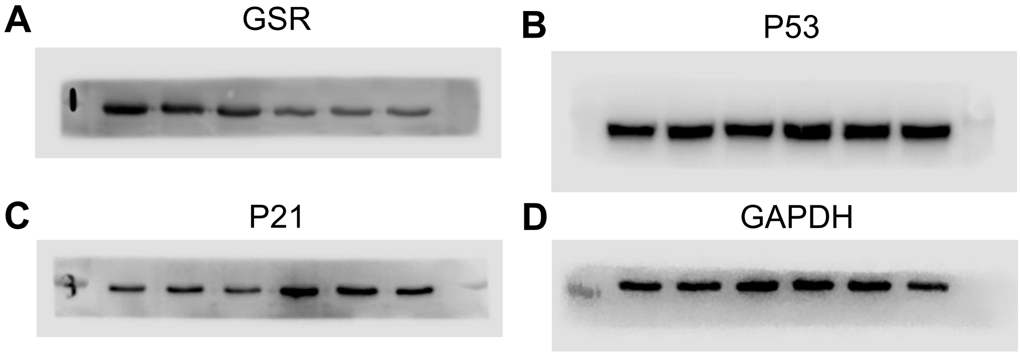

Figure S12. The original WB images of Figure 2G.

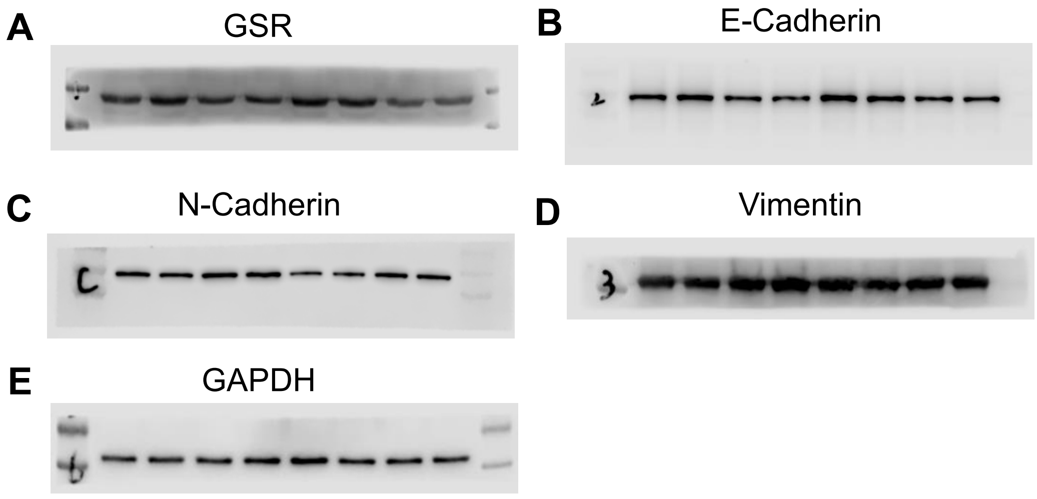

Figure S13. The original WB images of Figure 3H.

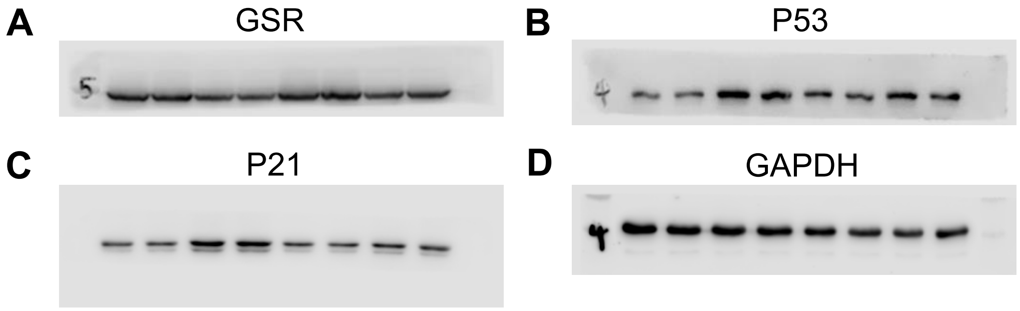

Figure S14. The original WB images of Figure 3J.

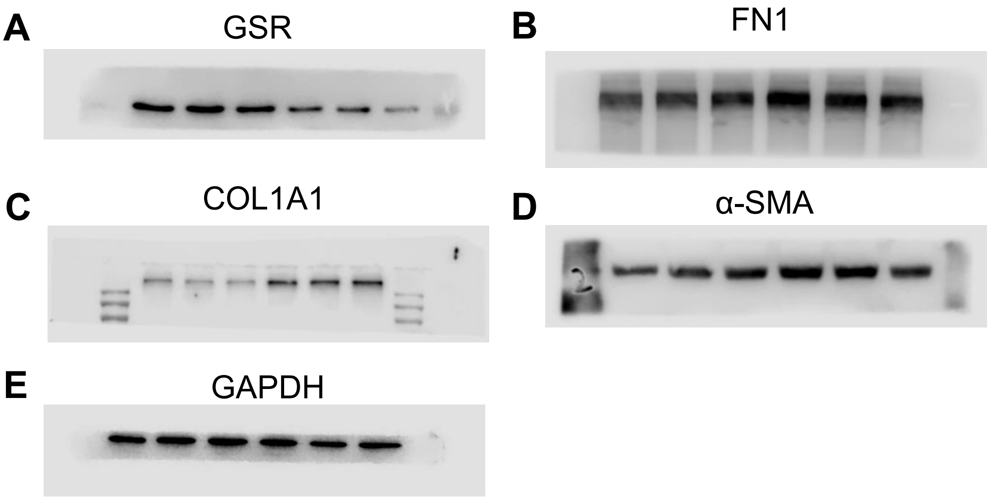

Figure S15. The original WB images of Figure 4A.

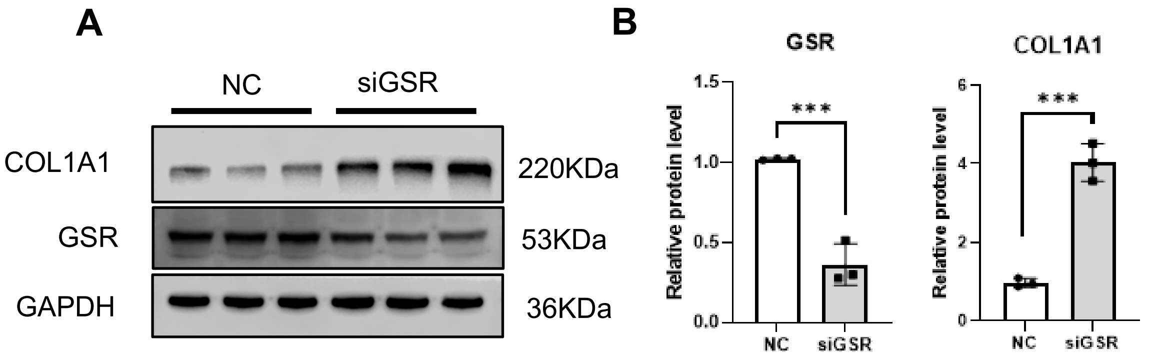

Figure S16. A. The protein levels of COL1A1, GSR and GAPDH in NC and siGSR groups. B. The gray level analysis of A, n = 3 replicates. ***$P < 0.001$, compared with the NC-siGSR group.

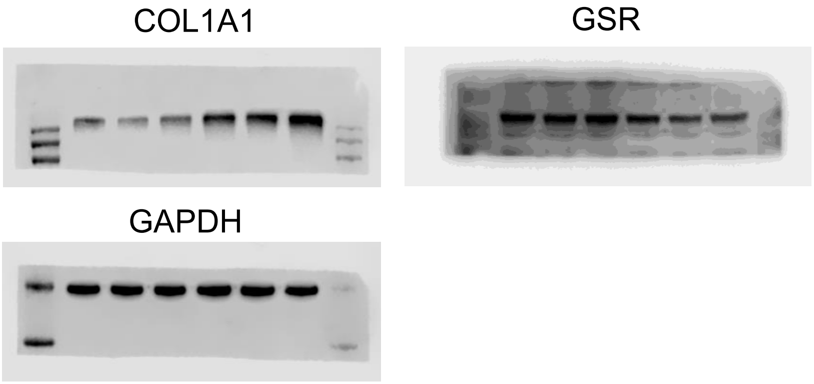

Figure S17. The original WB images of Figure S9A.

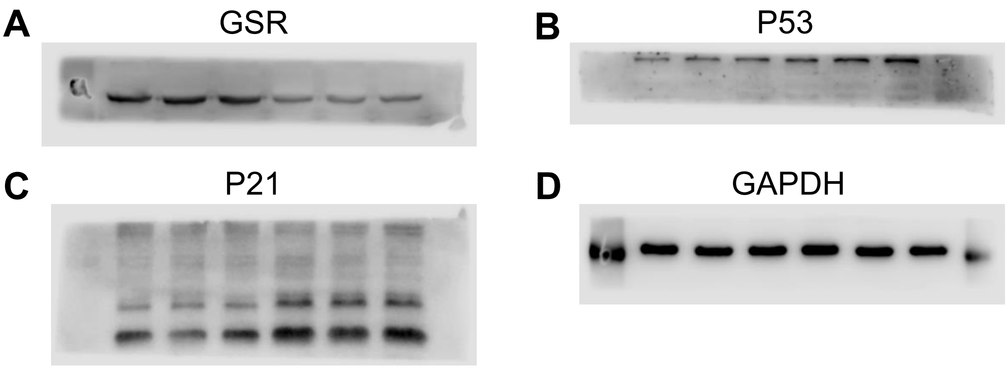

Figure S18. The original WB images of Figure 4G.

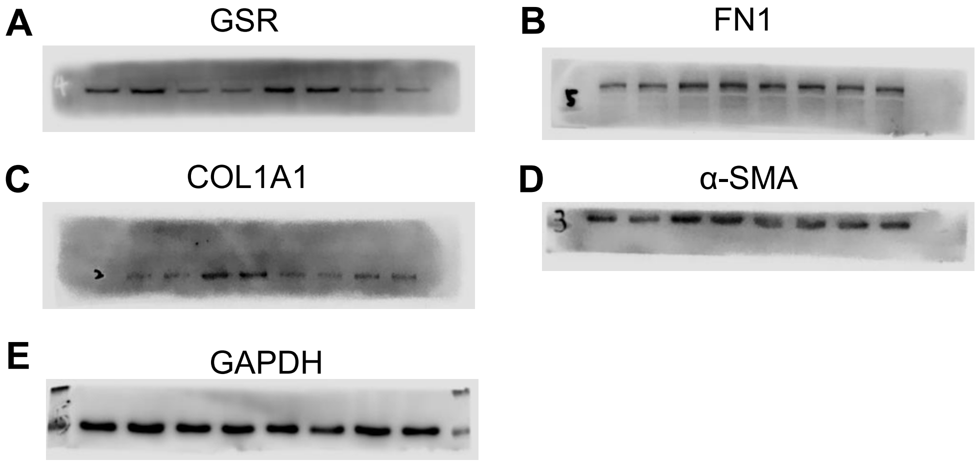

Figure S19. The original WB images of Figure 5G.

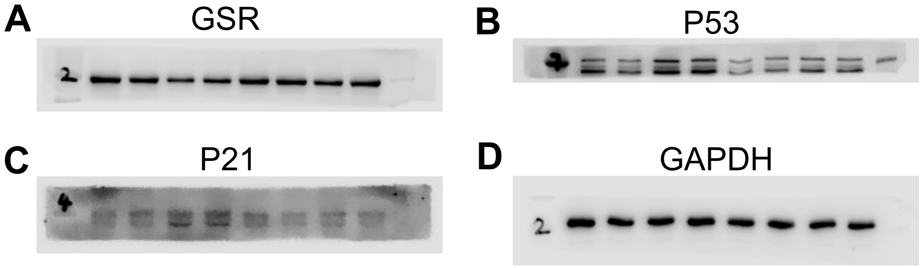

Figure S20. The original WB images of Figure 5K.

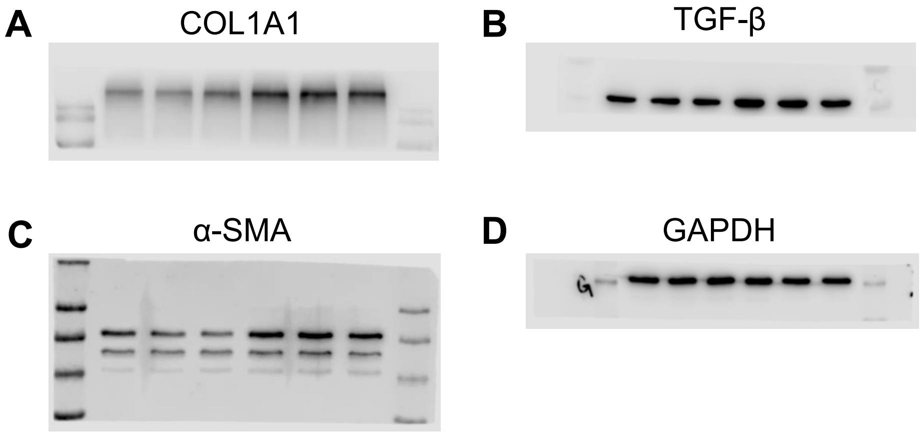

Figure S21. The original WB images of Figure 6A.

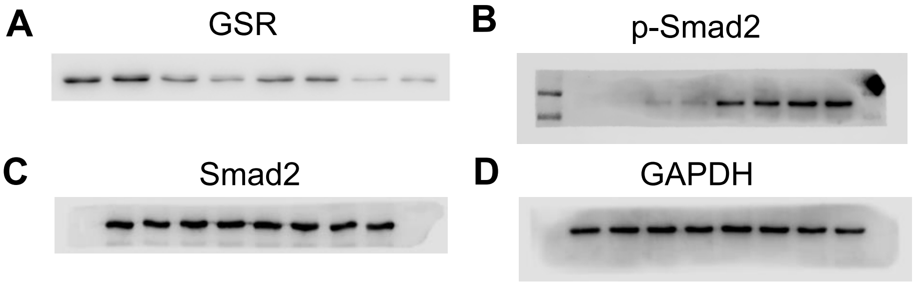

Figure S22. The original WB images of Figure 6D

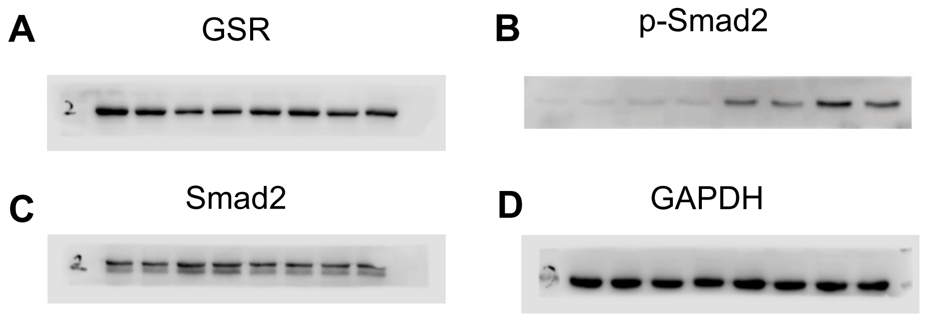

Figure S23. The original WB images of Figure 6F

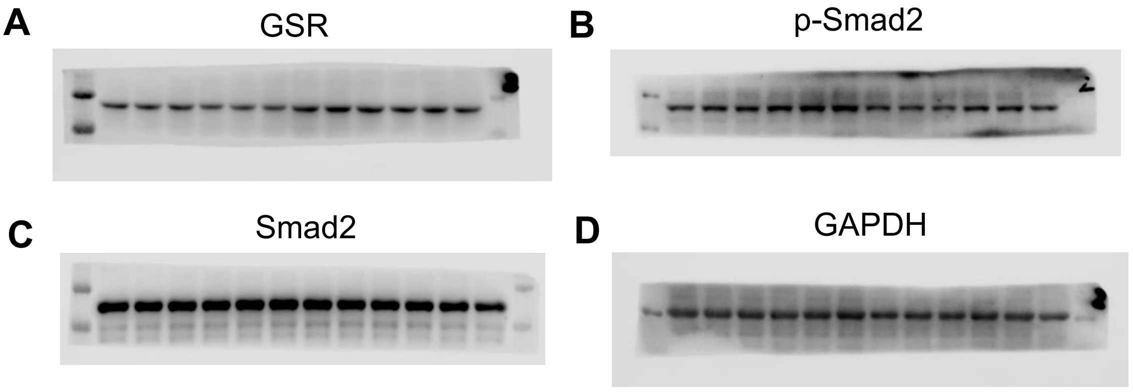

Figure S24. The original WB images of Figure 6L

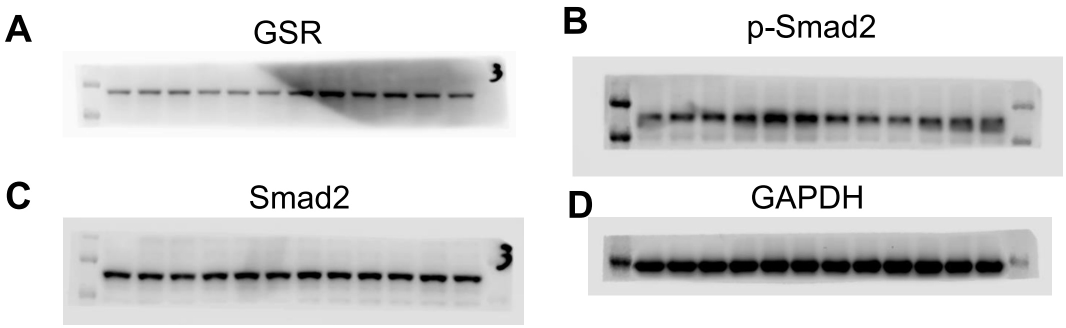

Figure S25. The original WB images of Figure 6N

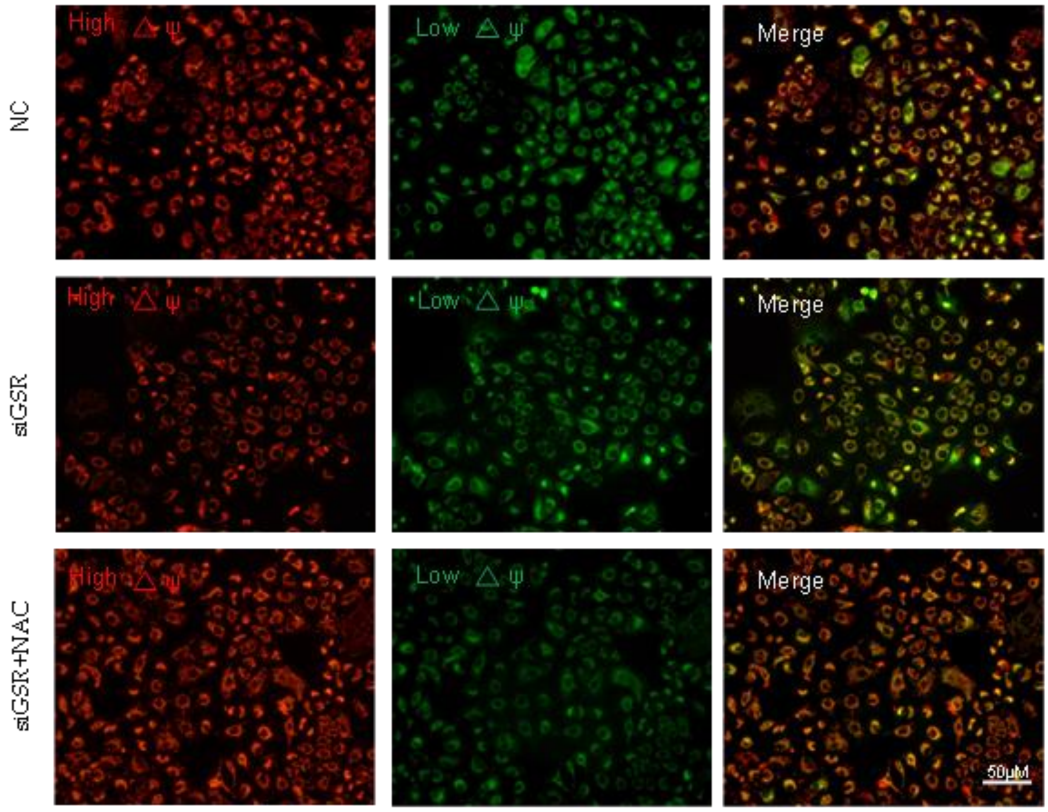

Figure S26. Detection of mitochondrial membrane potential via JC-10 staining. These results suggest that GSR knockdown impaired mitochondrial membrane potential, whereas the addition of NAC restored it.
